# Ancestry of the Timorese: age-related macular degeneration associated genotype and allele sharing among human populations from throughout the world

**DOI:** 10.3389/fgene.2015.00238

**Published:** 2015-07-09

**Authors:** Margaux A. Morrison, Tiago R. Magalhaes, Jacqueline Ramke, Silvia E. Smith, Sean Ennis, Claire L. Simpson, Laura Portas, Federico Murgia, Jeeyun Ahn, Caitlin Dardenne, Katie Mayne, Rosann Robinson, Denise J. Morgan, Garry Brian, Lucy Lee, Se J. Woo, Fani Zacharaki, Evangelia E. Tsironi, Joan W. Miller, Ivana K. Kim, Kyu H. Park, Joan E. Bailey-Wilson, Lindsay A. Farrer, Dwight Stambolian, Margaret M. DeAngelis

**Affiliations:** ^1^Ophthalmology and Visual Sciences, John A. Moran Eye Center, University of UtahSalt Lake City, UT, USA; ^2^National Children's Research Centre, Our Lady's Children's HospitalDublin, Ireland; ^3^Academic Centre on Rare Diseases, School of Medicine and Medical Science, University College DublinDublin, Ireland; ^4^The Fred Hollows Foundation New ZealandAuckland, New Zealand; ^5^National Centre for Medical Genetics, Our Lady's Children's HospitalDublin, Ireland; ^6^Computational and Statistical Genomics Branch, National Human Genome Research Institute, National Institutes of HealthBaltimore, MD, USA; ^7^Institute of Population Genetics, The National Research CouncilSassari, Italy; ^8^Department of Ophthalmology, Seoul National University College of MedicineSeoul, South Korea; ^9^Department of Ophthalmology, Seoul Metropolitan Government Seoul National University Boramae Medical CenterSeoul, South Korea; ^10^London School of Hygiene and Tropical Medicine, University of LondonLondon, UK; ^11^Department of Ophthalmology, Seoul National University Bundang HospitalSeoungnam, South Korea; ^12^Department of Ophthalmology, University of Thessaly School of MedicineLarissa, Greece; ^13^Retina Service and Ophthalmology, Massachusetts Eye and Ear Infirmary, Harvard Medical SchoolBoston, MA, USA; ^14^Departments of Medicine, Ophthalmology, Neurology, Epidemiology, and Biostatistics, Boston University Schools of Medicine and Public HealthBoston, MA, USA; ^15^Department of Ophthalmology, University of PennsylvaniaPhiladelphia, PA, USA

**Keywords:** population genetics, ancestry, age-related macular degeneration, complex disease, and epidemiology

## Abstract

We observed that the third leading cause of blindness in the world, age-related macular degeneration (AMD), occurs at a very low documented frequency in a population-based cohort from Timor-Leste. Thus, we determined a complete catalog of the ancestry of the Timorese by analysis of whole exome chip data and haplogroup analysis of SNP genotypes determined by sequencing the Hypervariable I and II regions of the mitochondrial genome and 17 genotyped YSTR markers obtained from 535 individuals. We genotyped 20 previously reported AMD-associated SNPs in the Timorese to examine their allele frequencies compared to and between previously documented AMD cohorts of varying ethnicities. For those without AMD (average age > 55 years), genotype and allele frequencies were similar for most SNPs with a few exceptions. The major risk allele of *HTRA1* rs11200638 (10q26) was at a significantly higher frequency in the Timorese, as well as 3 of the 5 protective *CFH* (1q32) SNPs (rs800292, rs2284664, and rs12066959). Additionally, the most commonly associated AMD-risk SNP, CFH rs1061170 (Y402H), was also seen at a much lower frequency in the Korean and Timorese populations than in the assessed Caucasian populations (C ~7 vs. ~40%, respectively). The difference in allele frequencies between the Timorese population and the other genotyped populations, along with the haplogroup analysis, also highlight the genetic diversity of the Timorese. Specifically, the most common ancestry groupings were Oceanic (Melanesian and Papuan) and Eastern Asian (specifically Han Chinese). The low prevalence of AMD in the Timorese population (2 of 535 randomly selected participants) may be due to the enrichment of protective alleles in this population at the 1q32 locus.

## Introduction

The population of Timor-Leste is afflicted by a very high prevalence of vision problems and ocular morbidity. In 2010, prevalence of blindness in adults aged ≥40 years was 3.6%, and cataract was the most common cause of blindness (Ramke et al., 2012). Although age-related macular degeneration (AMD) is the leading cause of blindness in developed countries, and the third leading cause world-wide, there is a low prevalence in Timor-Leste (Ramke et al., 2012). We therefore hypothesized that the genetic makeup of the Timorese may be enriched for protective variants.

The Democratic Republic of Timor-Leste (also known as East Timor), is a Southeast Asian country, inhabited by an ethnically diverse population which has not been extensively genetically characterized. Previously, two studies showed ~6-8% allele frequency differences for Y-chromosome short tandem repeat (Y-STR) markers between and among East Timorese and other neighboring populations (Kayser et al., 2001; Souto et al., 2006). Previous ancestry studies of Southeast Asian populations considered only mitochondrial DNA (mtDNA) markers (e.g., Melton et al., 1998; Hill et al., 2007; Tabbada et al., 2010) Y-STRs (e.g., Kayser et al., 2001; Karafet et al., 2010; Zhong et al., 2011; Van Oven et al., 2012), or both (e.g., Kayser et al., 2003; Hurles et al., 2005; Mona et al., 2009; Stoneking and Delfin, 2010). These studies consistently showed a preponderance of Y haplogroups O and M, and mtDNA haplogroups M, B, and F, although many other haplogroups are represented in Southeast Asia at lower frequencies.

We set out to characterize a sample population from Timor-Leste using a more comprehensive panel of both Y-STRs and mitochondrial DNA markers than had been previously reported, and data from a whole exome chip. Additionally, we used these data along with genotypes from 20 disease AMD associated single nucleotide polymorphisms (SNPs) to offer an explanation for the low frequency of AMD among Timorese.

## Materials and methods

### Timorese cohort: recruitment and assessment

The study protocol was reviewed and approved by the Institutional Review Board at the University of Utah and by the Timor Ministry of Health and conformed to the tenets of the Declaration of Helsinki. Participants were enrolled in this study after giving informed consent verbally and/or in writing before all data collection and examinations. Communications occurred in Tetum, the local language, or another language, depending on the participant's preference as previously described (Ramke et al., 2012). Six hundred and three subjects were recruited from Timor-Leste via a population-based cross-sectional survey using multistage cluster random sampling that has been described previously (Ramke et al., 2012). Briefly, individuals were phenotyped by external and intraocular examinations by a trained specialist (GB and JR). This included magnified assessment of the anterior and indirect ophthalmoscopy of the dilated posterior segment as previously described (Ramke et al., 2012). Both blood samples and epidemiological information were available for 535 subjects. Leukocyte DNA was purified by using standard phenol chloroform extraction methods, the DNAzol (Invitrogen) extraction protocol, or the QIAamp DNA Blood Maxi Kit (Qiagen). HbA1C levels were measured using a Roche/Hitachi COBAS C-311 instrument with reagent Tina-quant Hemoglobin A1c Gen.2. for each of the participants to rule out any diabetic disease.

### Ancestry of the timorese

We sequenced the Hypervariable I and II region of the mitochondrial genome of each individual. Reactions were prepared in 25 μl volume as follows: 2.5 μl of 10X PCR Rxn Buffer (Invitrogen), 0.5 μl of 10mM dNTP Mix (Invitrogen), 0.75 μl of 50 mM MgCl_2_(Invitrogen), 1 μl of each 10 μM PCR primer (mt-F15971 and mt-R484) (Integrated DNA Technologies Incorporated), 1.25 μl of dimethyl sulfoxide (Sigma), 0.2 μl of 5 units/μl Taq DNA polymerase (Invitrogen), and 10 μl of 2 ng/μl template DNA. Amplification reactions were carried out in a BIORAD MyCycler thermal cycler with the following protocol: initial denaturation 94°C for 2 min, 38 cycles at 94°C for 15 s, 54°C for 30 s, and 72°C for 1 min and 15 s, and a final extension at 72°C for 5 min. We digested 5 μl of the post-PCR product with 2 μl of ExoSAP-IT (USB), according to the manufacturer's protocol. We added 1 μl of each forward 10 μM sequencing primer (mt-F15 and mt-F15971) to 7 μl of digested post-PCR product (Integrated DNA Technologies Incorporated), and 1 μl of 10 μM reverse sequencing primer (mt-R141) to 7 μl of digested post-PCR product (Integrated DNA Technologies Incorporated). Sequences were analyzed using Sequencher 5.0 (Gene Codes Corporation) independently by two researchers to ensure that mutations were true events rather than mere sequencing errors.

We assessed the degree of Y-chromosome variation among the Timorese males represented in our population sample using 17 short tandem repeats (STRs) loci (DYS19, DYS385a/b, DYS389I/II, DYS390, DYS391, DYS392, DYS393, DYS437, DYS438, DYS439, DYS448, DYS456, DYS458, DYS635, and Y-GATA H4) using the AmpF*l*STR Yfiler Amplification Kit (Applied Biosystems) using the manufacturer's recommendations. DNA was amplified in a GeneAMp PCR System 9700 equipped with silver blocks and DNA fragment separation and detection was carried out on an ABI Prism 3100 Genetic Analyzer (Applied Biosystems). Genescan500 LIZ (ABI) was utilized as an internal size standard. Data were analyzed with an allelic ladder as well as positive and negative controls using GeneMapper® 3.2 (ABI) and were double-checked manually for accuracy.

Haplogroup assignation for the Y-STR markers was carried out with World Haplogroup & Haplo-I Subclade Predictor (http://members.bex.net/jtcullen515/haplotest.htm) and confirmed using Althey's Haplogroup Predictor (http://www.hprg.com/hapest5/). The two identifying SNPs for haplogroups O3 (M122 and P198) and O2 (P31 and M268) were confirmed using a Taqman assay and direct sequencing, respectively [following Van Oven (Van Oven et al., 2012) for primer design]. Haplogroup assignment for the Hypervariable I and II region of the mitochondrial genome was determined using MitoTool (www.mitotool.org) and confirmed using HaploGrep (haplogrep.uibk.ac.at).

Genotyping using the Illumina HumanExome array version 1.1 chip was also performed at the Center for Inherited Disease Research (CIDR) as part of a refractive error consortium study. This chip not only includes >240,000 SNPs including 30,000 custom probes but most importantly 3468 Ancestry informative markers. CIDR laboratory standard quality control procedures were applied to the entire dataset prior to release of the genotype data from the laboratory. Once sample collection was finalized, samples were plated controlled-randomized to avoid spurious associations due to plate effects. This QC included the following: Blind duplicates and HapMap controls were distributed across plates for concordance checking. Samples with suspected mixtures or unusual X and Y patterns or gender mismatch were identified and dropped before release of genotype data from the CIDR lab. SNP clustering was performed on all SNPs in the project and SNP genotypes with genotype quality (GC) score less than 0.15 were recoded as missing genotypes. Autosomal SNPs with less than 85% call rate, cluster separation of less than 0.3 and heterozygote rate greater than 80% were dropped prior to release of genotype data from the laboratory. A subset of SNPs was also manually reviewed that included all Y, XY pseudoautosomal and mitochondrial SNPs and other various circumstances.

After receiving data from CIDR, additional quality control measures were applied. Sex discrepancies were calculated in PLINK (Purcell et al., 2007) (http://pngu.mgh.harvard.edu/~purcell/plink/) and samples which did not appear sufficiently matched to their recorded sex were dropped. Any unexpected duplicate samples were identified using PREST-PLUS (http://utstat.toronto.edu/sun/Software/Prest/) and one of the duplicate pair dropped. SNPs with >1 errors in blind duplicates or HapMap controls were dropped and SNPs with >1 Mendelian error after correction of pedigree relationships were also removed. Testing for batch effects was performed using a homogeneity test of minor allele frequency for each SNP on each plate compared to all other plates (Pluzhnikov et al., 2008, 2010). These statistics were then averaged over all SNPs to determine how the plates deviate from each other in PLINK. Heterozygosity rates across samples were checked and outlier samples excluded. Examination of samples for chromosomal abnormalities was performed and problematic samples were identified. Autosomal SNPs with sex difference in allelic frequency >0.2, sex difference in heterozygosity > 0.3 were also excluded. Monomorphic variants were also excluded.

Resulting genotype data were then merged with the samples from the Human Genome Diversity Project (HGDP, http://www.hagsc.org/hgdp/index.html) using—merge in PLINK, ensuring that all overlapping SNPs were on the same strand, and removing all AT and CG SNPs. Assessment of population sub-structure in this resulting merged HGDP-Timor dataset was performed using principal components analysis with Eigensoft (Patterson et al., 2006; Price et al., 2006) (http://www.hsph.harvard.edu/alkes-price/software/), Admixture version 1.2 (Alexander et al., 2009) (http://www.genetics.ucla.edu/software/admixture/download.html), and Ancestry Mapper (Magalhaes et al., 2012) (http://cran.r-project.org/web/packages/AncestryMapper/index.html). Ancestry Mapper is a program that uses SNPs to create a unique individual genetic identifier and inform ancestry by comparing that identifier to those of individuals with known genetic ancestry. For this study, the Timorese individuals were compared to the 51 populations included in the HGDP. This includes individuals a world wide range of populations covering all continents including Africa, Europe, the Middle East, South and Central Asia, East Asia, Oceania and the Americas (Cann et al., 2002; Rosenberg et al., 2002, 2005) and is a widely used dataset to study genetics of populations (Cavalli-Sforza et al., 1991; Tishkoff et al., 2009; International HapMap 3; Li et al., 2008; López Herráez et al., 2009; International HapMap 3 Consortium et al., 2010). Using all three methods, which rely on different algorithms, assures the consistency and cross-validation of the results.

### AMD-associated SNPs

To investigate the genetic basis for the low prevalence of AMD in this cohort, we genotyped 20 previously reported AMD-associated SNPs to examine their allele frequencies in the Timorese and other populations of different ethnicities. This included six SNPs from the *CFH* (1q32) region (*CFH* SNPs rs800292, rs16840422, rs1061170, rs12144939, and rs2284664, and *CFHR2* rs3790414) (Klein et al., 2005; Li et al., 2006; Zhang et al., 2008; Sivakumaran et al., 2011), along with the *CFHR1–3*Δ change (Hughes et al., 2006), 3 *ROBO1* (3p12) SNPs (rs1387665, rs4513416, and rs9309833) (Jun et al., 2011), 2 SNPs from the 6p21 region where *C2* resides (*C2* rs9332739 and rs547154) (Gold et al., 2006), 6 SNPs from *ARMS2/HTRA1* (10q26) region (*ARMS2* rs10490924 and rs10664316, and *HTRA1* rs11200638, rs2672598, rs1049331, and rs2293870) (Dewan et al., 2006; Yang et al., 2006; Deangelis et al., 2008), and 3 *RORA* (15q22) SNPs (rs12900948, rs8034864, and rs730754) (Schaumberg et al., 2010; Silveira et al., 2010; Jun et al., 2011). These SNPs were also genotyped in three diverse and carefully AMD-phenotyped populations including a family-based cohort from New England (*n* = 657) (DeAngelis et al., 2004; Silveira et al., 2010), a case-control cohort from Central Greece (*n* = 436) (Silveira et al., 2010), and a case-control cohort recruited from Seoul National University Bundang Hospital (*n* = 818) (Fritsche et al., 2013).

All *CFH, CFHR2, C2, ROBO1, ARMS2, HTRA1* (rs11200638 and rs2672598), and *RORA* SNPs were genotyped using a combination of pre-designed and Custom Taqman SNP Genotyping Assays (Applied Biosystems). Each assay was run in a 15 ul reaction containing 2× Taqman GTXpress master mix, 40× or 80× probe, and 10 ng of DNA. Thermal cycling was performed according to the manufacturer's protocol. The ABI 7500 Real-Time PCR System, with the accompanying software, was used to analyze the genotypes. Direct sequencing was used to genotype *HTRA1* SNPs (rs1049331 and rs2293870) using previously reported oligonucleotide primers and methods (Deangelis et al., 2008). For sequencing reactions, PCR products were digested according to the manufacturer's protocol using ExoSAP-IT (USB). DNA sequencing was performed at the University of Utah DNA Sequencing Core Laboratory. Electropherograms were read independently by two evaluators without knowledge of the subject's disease status. *CFHR1–3Δ* status was determined directly in the Timorese cohort using a pre-designed Taqman copy number assay (Applied Biosystems). Each sample was run in triplicate in 20 ul reactions containing 2× Taqman GTXpress master mix, 20× probe, 20× RNaseP, and 20 ng of genomic DNA. Data was analyzed using CopyCaller v2.0 software. *CFHR1–3Δ* status was compared to the T allele of rs12144939 to determine its tagging validity.

Allele frequencies in each cohort were calculated for normal subjects, or those without any evidence of documented AMD. Linkage disequilibrium among the genotyped SNPs was calculated using only those without any signs of AMD in Haploview (http://www.broadinstitute.org/scientific-community/science/programs/medical-and-population-genetics/haploview/haploview).

## Results

### Subjects

Six hundred and 3 subjects from Timor-Leste were examined as part of this study. Of these 603 subjects, both blood samples and epidemiological information were available for 535 subjects. Of these 535 subjects, 267 were males and 268 were females. Average HbA1c was 5.57 ± 0.71 for this cohort. A diagnosis of early AMD was given to two subjects from the Timor population: a 62 year old female and a 75 year old male. The average age of the study participants without AMD was 55.12 years (range 40–94). Subject characteristics of all populations examined in this study are shown in Table 1.

**Table 1:** **Characteristics of subjects without AMD**.

**Characteristic**	**NESC**	**Greek**	**Korean**	**Timor**
n	198	213	384	533
Males (% total)	87 (43.9%)	100 (46.9%)	194 (50.5%)	268 (50.1%)
Age (range)	75.45 (50–94)	73.78 (48–95)	68.46 (50–87)	55.12 (40–94)

### Ancestry analysis

**Figure 1:**
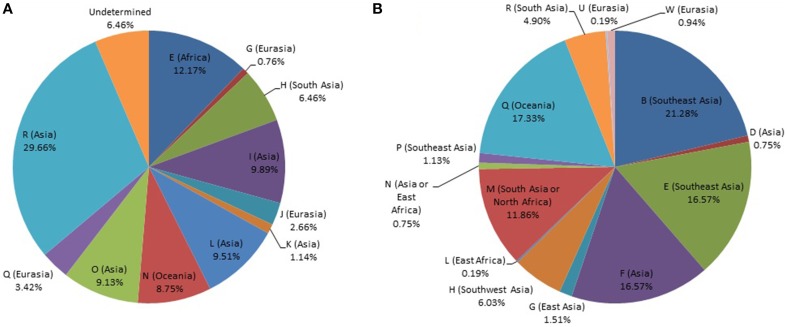
**Distribution of world haplogroups in the Timorese sample. (A)** Distribution of the Y-STR haplogroups among males. **(B)** Distribution of the mtDNA haplogroups. Populations are shown according to color.

**Figure 2:**
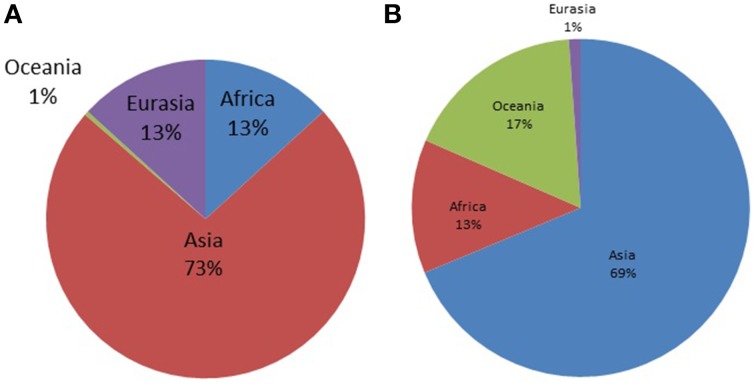
**Distribution of world haplogroups in the Timorese sample by continent. (A)** Shows the distribution of the Y-STR haplogroups among the 248 Timorese males, grouped by the continent each haplogroup originated from. **(B)** Shows the distribution of the mtDNA haplogroups among the 535 Timorese subjects, grouped by the continent each haplogroup originated from.

The Y-STR analysis yielded genotypes for 265 of the 267 Timorese males. Using the publicly available databases World Haplogroup & Haplo-I Subclade Predictor (http://members.bex.net/jtcullen515/haplotest.htm) and Althey's Haplogroup Predictor (http://www.hprg.com/hapest5/), 11 haplogroups were assigned to 248 of these subjects (Figure 1A). The origin of the haplogroups is mostly Asia (73%), followed by Africa and Eurasia (13% each), and a small percent Oceana (1%) (Figure 2A).

MtDNA genotypes were determined for all 535 Timorese subjects using the publicly available reference data and are distributed among 14 haplogroups (Figure 1B). The haplogroups distribution shows that the origin of the Timorese males is mostly Asian (69%), followed by Oceanic (17%), African (13%), and a small percent Eurasian (1%), (Figure 2B). Given the within-group diversity present, it is likely that either multiple migrations or a few migrations of distantly related male individuals from Asia and other parts of the Old World have populated the islands, which is consistent with pre-historical and historical events.

**Figure 3:**
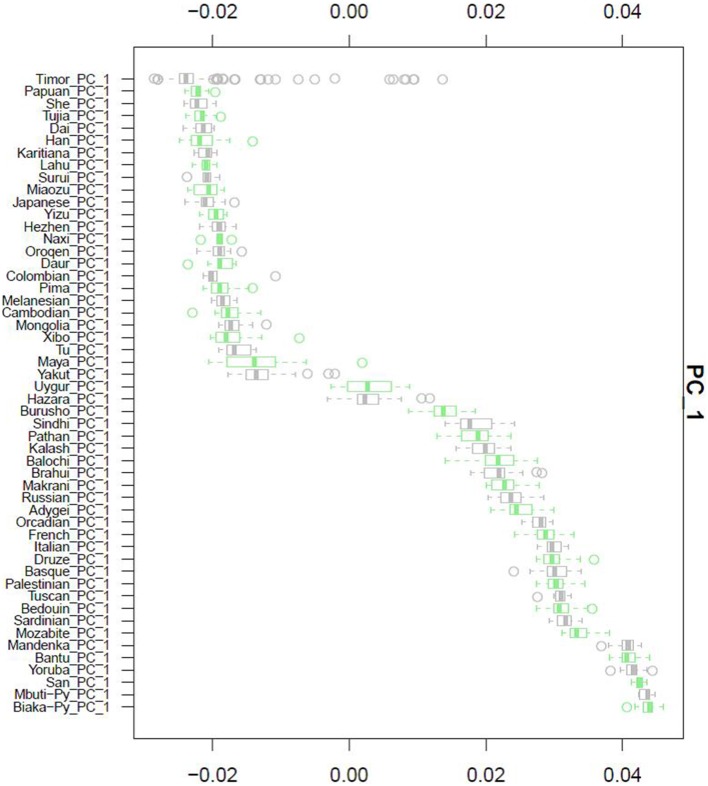
**Principal Component Analysis**. Principal component 1, PC1, as calculated for each of the 51 HGDP references and compared to the Timorese individuals investigated in this study. The Y-axis shows each of the reference populations. The X-axis shows the calculated PC1 value for each population.

**Figure 4:**
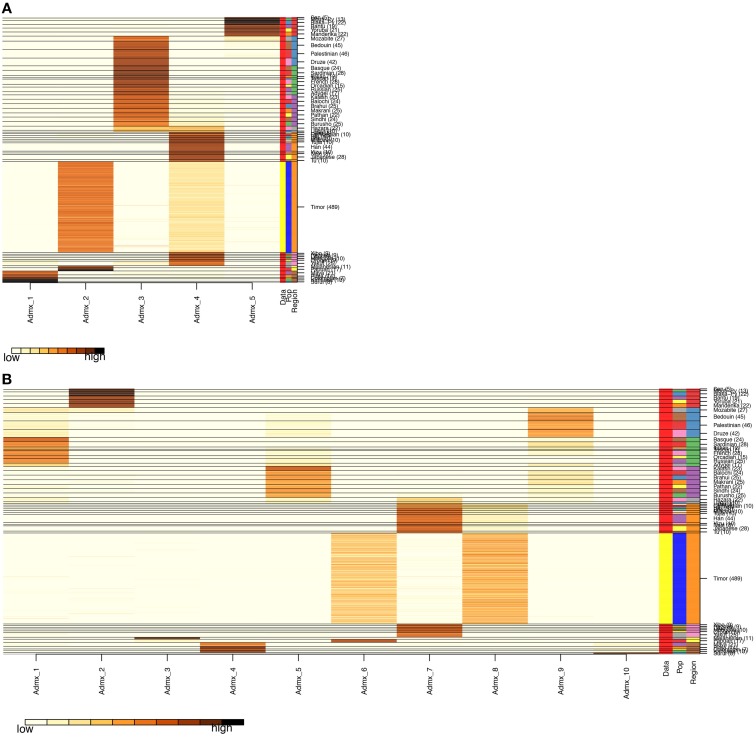
**Admixture Analysis**. The distribution of individuals of each population (the 51 HGDP references and the Timorese subjects), per cluster. The Y-axis shows each of the reference populations and the number of subjects within each population. The X-axis shows the clusters. The darker the color corresponding to each cluster indicates a large number of subjects assigned to that cluster. **(A)** Shows the distribution when the number of clusters, k, is 5 and **(B)** shows *k* = 10.

**Figure 5:**
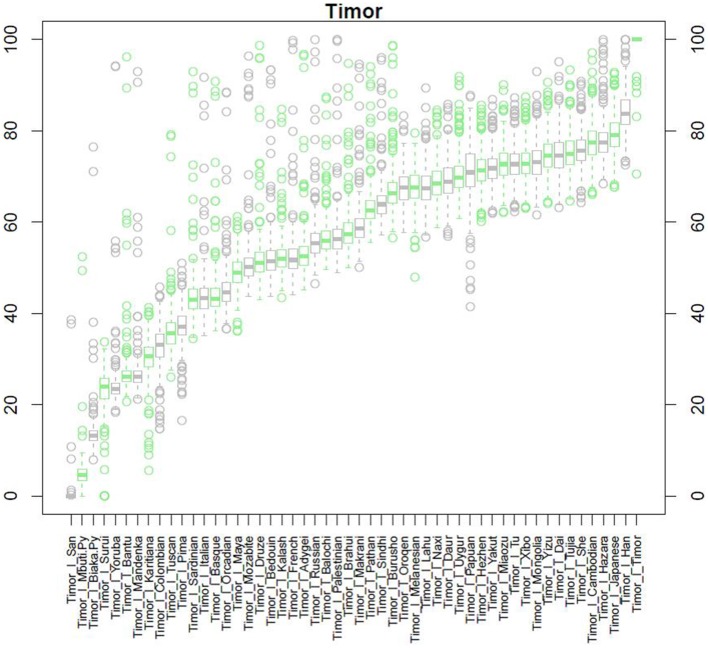
**Ancestry Mapper Analysis**. This figure shows the comparison of the Timorese indivuduals to the 51 HGDP reference populations, as calculated by Ancestry Mapper. The Y-axis shows the percentage of the Timorese individuals that are most closely related to each reference population. The X-axis shows each population the Timorese samples are compared to.

After QC and data cleaning, genotypes from 253,405 SNPs were available for 489 Timorese subjects with a call rate of >98% and were included in the subsequent analyses. There were 11,064 common SNPs between the HGDP dataset and the Timorese dataset used for subsequent ancestry analyses. Principal components analysis showed that the first principal component, PC1, was most similar between the Timorese and Papuans, followed by the She, Tujia, Dai, and Han Chinese (mean PC1 ~ −0.02; Figure 3). Using admixture, we have used several number of groupings and have found that *K* = 5 organizes the populations into the known ancestral populations, i.e., African, Indo-European, Oceania, Amerindian and Asian. Those global ancestry components allow us to separate HGDP, a dataset with worldwide coverage into their well-established continental differences since the known populations are correctly assigned. Where *k* = 5, the Timorese samples were showing the greatest proportion of Admix group 2, followed by a smaller proportion of Admix group 4 (Figure 4A). Individuals with the highest values for Admix group 2 are mostly of Oceanic populations, such as the Melanesian and Papuan samples, which are geographically close to Timor-Leste. Individuals with high values for Admix group 4 are from Eastern Asia, including Japanese and several Chinese populations. By increasing the number of ancestral assignments (k) to 10, there is a clearer separation of the Timorese samples, in that they show a closer relationship with the Papuan samples, and not as much the Melanesian (Figure 4B). Using Ancestry Mapper, the largest percentage of the Timorese was assigned to the Han Chinese reference population (~85%, Figure 5).

Only two individuals in our cohort were affected by AMD. The first was a 62 year old female, whose mitochondrial haplogroup is F3b1 (thus implying maternal Southeast Asian ancestry) and whose principal component 1 was very close to the average of the group and the Papuan reference (PC1 = -0.0250). Using admixture, this subject was shown to be Oceanic (*k* = 5) and when *k* = 10, this individual showed the most similarity to Han Chinese (50.5%) and also Papuan (43.3%). Finally, Ancestry Mapper showed that aside from the Timorese reference group, this individual was given the highest score to the Han Chinese reference group (79.0%), followed by the Cambodian reference group (75.9%). The second AMD individual was a 75 year old male, who belongs to Y-haplogroup H (thus implying a likely South Asian paternal origin) and mitochondrial haplogroup B4b1 (thus implying maternal Southeast Asian ancestry). This subject was shown by principal components to be closest to the Papuan reference (PC1 = −0.0217) and by Admixture to be Oceanic when *K* = 5 but a split of Han Chinese and Papuan when *K* = 10 (48.3 and 48.0%, respectively). Finally, using Ancestry Mapper, after the Timorese reference group, this individual was given the highest score to the Han Chinese reference group (81.1%), followed by the Hazara reference (77.3%).

### AMD-associated SNPs

The frequency of alleles of the 20 genotyped SNPs was statistically significantly different between the Timorese and other genotyped cohorts for the majority of the SNPs investigated. Specifically, statistically significantly differences were observed in 11 SNPs between the Timorese and the NESC cohort, 16 SNPs between the Timor and Greek cohorts, and 18 SNPs between the Timor and Korean cohorts (*p* < 0.05, Table 2). For all cohorts examined, the most statistically significant differences in allele frequencies were observed in the *CFH* (1q32) region, followed by the *ARMS2/HTRA1* (10q26) region. The two most consistently AMD-associated SNPs, *CFH* rs1061170 (Y402H) and *ARMS2* rs10490924 (A69S) were not statistically different between the Timorese and all cohorts. The alleles of Y402H were significantly differently distributed between the Timor and Caucasian cohorts (NESC and Greek) but not the Koreans. Specifically, the risk allele, C, was significantly less frequent in the Timorese than in the Caucasians. The alleles of A69S were statistically different between the Timorese and the Greeks and Koreans but not the Caucasians. Specifically, the risk allele, T, was significantly higher in the Timorese than the Greeks but lower than the Koreans.

**Table 2:** **AMD-Associated SNP Allele Frequencies**.

**SNP**	AMD Effect^*^	**Chr**.	**Position**	**Alleles**	**Timor**	**NESC**	***p*-value**	**Greek**	***p*-value**	**Korean**	***p*-value**
*CFH* rs800292	A is Protective	1	194908856	G/A	*A* = 0.688	*A* = 0.205	<0.0001	*A* = 0.220	<0.0001	*A* = 0.397	<0.0001
*CFH* rs16840422	T is Protective	1	194919457	C/T	*T* = 0.155	*T* = 0.131	0.2550	*T* = 0.110	0.0237	*T* = 0.083	<0.0001
*CFH* rs1061170	C is Risk	1	194925860	T/C	*C* = 0.067	*C* = 0.429	<0.0001	*C* = 0.373	<0.0001	*C* = 0.069	0.8601
*CFH* rs12144939	T is Protective, tags for CFHR1-3Δ	1	194965568	G/T	*T* = 0.146	*T* = 0.170	0.2654	*T* = 0.213	0.0018	*T* = 0.053	<0.0001
*CFH* rs2284664	T is Protective	1	194969148	C/T	*T* = 0.613	*T* = 0.197	<0.0001	*T* = 0.207	<0.0001	*T* = 0.364	<0.0001
*CFHR2* rs3790414	A is Protective	1	195186922	T/A	*A* = 0.764	*A* = 0.240	<0.0001	*A* = 0.250	<0.0001	*A* = 0.683	0.0002
*ROBO1* rs1387665	T is Risk	3	79512501	C/T	*T* = 0.553	*T* = 0.497	0.0706	*T* = 0.493	0.0411	*T* = 0.380	<0.0001
*ROBO1* rs4513416	A is Protective	3	79573493	G/A	*A* = 0.346	*A* = 0.413	0.0191	*A* = 0.407	0.0305	*A* = 0.468	<0.0001
*ROBO1* rs9309833	C is Risk	3	79894409	T/C	*C* = 0.178	*C* = 0.124	0.0159	*C* = 0.172	0.7890	*C* = 0.308	<0.0001
*C2* rs9332739	C is Protective	6	32011783	G/C	*C* = 0.006	*C* = 0.035	<0.0001	*C* = 0.050	<0.0001	*C* = 0.024	0.0011
*C2* rs547154	T is Protective	6	32018917	G/T	*T* = 0.166	*T* = 0.073	<0.0001	*T* = 0.070	<0.0001	*T* = 0.101	<0.0001
*ARMS2* rs10490924	T is Risk	10	124204438	G/T	*T* = 0.336	*T* = 0.311	0.3680	*T* = 0.237	0.0002	*T* = 0.398	0.0064
*ARMS2* rs10664316	- is Protective	10	124206375	delAT	- = 0.331	- = 0.396	0.0223	- = 0.415	0.0023	- = 0.358	0.2431
*HTRA1* rs11200638	A is Risk	10	124210534	G/A	*A* = 0.635	*A* = 0.303	<0.0001	*A* = 0.226	<0.0001	*A* = 0.405	<0.0001
*HTRA1* rs2672598	C is Risk	10	124210672	T/C	*C* = 0.523	*C* = 0.492	0.3028	*C* = 0.534	0.7109	*C* = 0.711	<0.0001
*HTRA1* rs1049331	T is Risk	10	124211260	C/T	*T* = 0.358	*T* = 0.306	0.0643	*T* = 0.231	<0.0001	*T* = 0.407	0.0320
*HTRA1* rs2293870	T and C are Risk	10	124211266	G/T/C	*T* = 0.355, *C* = 0.001	*T* = 0.308, *C* = 0.089	0.1489	*T* = 0.226, *C* = 0.164	0.2251	*T* = 0.407, *C* = 0.000	0.0288
*RORA* rs12900948	G is Protective	15	59227963	A/G	*G* = 0.413	*G* = 0.410	0.9246	*G* = 0.502	0.0020	*G* = 0.580	<0.0001
*RORA* rs8034864	A is Risk	15	59233645	C/A	*A* = 0.417	*A* = 0.209	<0.0001	*A* = 0.290	<0.0001	*A* = 0.587	<0.0001
*RORA* rs730754	G is Protective	15	59238628	A/G	*G* = 0.420	*G* = 0.397	0.4331	*G* = 0.468	0.0941	*G* = 0.618	<0.0001

p-values shown are those comparing allele frequencies between each cohort and Timorese.

* *AMD References: CFH region (1q32) (Hageman et al., 2005; Haines et al., 2005; Klein et al., 2005; Hughes et al., 2006; Li et al., 2006; Zhang et al., 2008; Sivakumaran et al., 2011); ROBO1 (3p12) (Jun et al., 2011); C2 (6p21 region) (Gold et al., 2006); ARMS2/HTRA1 (10q26) (Dewan et al., 2006; Yang et al., 2006; Deangelis et al., 2008); and RORA (15q22) (Schaumberg et al., 2010; Silveira et al., 2010; Jun et al., 2011)*.

Notably, the neither of the two subjects with early AMD in this Timorese cohort had risk alleles at either of the two major risk loci, *CFH* Y402H (rs1061170) or *ARMS2* A69S (rs10490924). Interestingly, the *CFH* SNP rs12144939, which has been shown to tag for the protective CFHR1-3Δ, was observed at a relatively similar frequency in each of the cohorts examined (MAF ~ 5–20%, with the Koreans having the lowest frequency), although it's tagging ability was only validated in the NESC cohort.

The linkage disequilibrium (LD) patterns among the genotyped SNPs showed differences between the four populations (Supplementary Table 1). On chromosome 1 where *CFH* resides, no haplotype blocks were observed for the NESC, Greek or Korean cohorts, while in the Timorese, a haplotype block encompassing 5 SNPs was defined by the Gabriel rule (Gabriel et al., 2002) (*CFH* rs16840422, rs1061170, rs2284664, and rs12144939). On chromosome 10, the LD patterns were similar between the Caucasian cohorts and between the Korean and Timorese cohorts. Specifically, in the Caucasian cohorts there was high LD (*r*^2^ > 0.8) among 3 pairs of SNPs, while in the Korean and Timorese cohorts there was high LD among 6 sets of SNPs. On 15q22, there was high LD among two *RORA* SNPs in all cohorts, while the Korean and Timorese cohorts also showed high LD among the third. No significant differences in LD were observed on chromosome 3 (*ROBO1*) or chromosome 6 (*C2*).

## Discussion

Utilization of the Timorese cohort provides a unique opportunity to study protection from a complex disease, AMD, in a geographically isolated population with low disease prevalence. AMD is the only complex disease for which 2 loci, *CFH* and *ARMS2/HTRA1*, explain a large proportion of risk (Edwards et al., 2005; Hageman et al., 2005; Haines et al., 2005; Klein et al., 2005; Dewan et al., 2006; Yang et al., 2006, p. 200; Swaroop et al., 2007; Deangelis et al., 2008; Fritsche et al., 2013). In addition, AMD risk is explained by many other loci which implicate several mechanisms and pathways underlying AMD pathophysiology (for review please see, Swaroop et al., 2009; Deangelis et al., 2011; Fritsche et al., 2013; Miller, 2013).

Other studies have shown that population structure and admixture can have strong confounding effects on the determination of genetic association analyses (e.g., Shtir et al., 2009). This is an important factor for which to account when studying the genetics of AMD, as there are inconsistencies in the distribution of AMD-related alleles and AMD prevalence among different world populations (e.g., Grassi et al., 2006; Mori et al., 2007; Nonyane et al., 2010). Although this is not meant to be an exhaustive literature review, studies have shown that the risk allele at Y402H (rs1061170) located in *CFH*, which is one of the most strongly associated markers to any complex disease [odds rations (ORs) >5 for the homozygous risk allele genotype, Klein et al., 2005], has a high frequency in Caucasians (Edwards et al., 2005; Hageman et al., 2005; Haines et al., 2005; Klein et al., 2005). These observations have been documented independently by different researchers who studied different populations, including the Japanese (Grassi et al., 2006; Mori et al., 2007), Chinese (Ng et al., 2008; Xu et al., 2008), and Koreans (Kim et al., 2008). On the other hand, Africans were shown to have comparatively high frequencies of the deleterious alleles, but low AMD prevalence (though not adjusted for life expectancy) (Friedman et al., 1999; Klein et al., 1999; Muñoz et al., 2000; Ziskind et al., 2008).

Our work has shown that the Timorese population represented by 535 phenotyped individuals exhibits an increased frequency of some protective genotypes (*CFH* rs800292, rs1684022, and rs2284664) and a decreased frequency of a major risk genotype (at *CFH* rs1061170), thus further confirming the implication of the two main loci on chromosome 1 and 10. However, the Timorese cohort exhibits a lower frequency of some protective alleles as compared to the other population(s) reported here (*CFH* rs12144939 and rs12066959; *HTRA1* rs2672598; *ARMS2* rs10664316), thus emphasizing the difficulty of identifying causative alleles implicated in the pathogenesis of complex diseases.

Studying the genetic variation in different populations where AMD prevalence and disease expression (i.e., phenocopy) varies is of paramount importance if we wish to untangle the relationship between genotype and phenotype, and genotype and environment. Additionally, comparing genotypes among populations can facilitate the identification of causal variants, a process that can be masked by the effect of linkage disequilibrium (LD). In a case where causal and non-causal variants are inherited together as part of one LD block, the identification of causal variants is difficult, as both causal and non-causal variants may have similar frequencies. Populations from different lineages have an increased chance of having lost some of the variants due to recombination, and may differ from other populations with diverse evolutionary histories. Finally, this study shows that ancestry plays a remarkable role in the risk of developing a disease, given differential exposure to selective stressors during the natural history of each population.

This ancestry analysis in the Timorese builds on the work that was performed by Souto et al. (2006), as the world haplogroup databases have grown in the past several years. We confirm the genetic diversity of this population found by Souto but add to the diversity with the addition of Oceanic groupings. The mtDNA analyses conducted in this study show that the Timorese populations represented in our sample cohort have a complex natural history. Since mtDNA is maternally inherited, it does not recombine, and has a high (but fairly constant) mutation rate, it allows us to study lineage-specific variation in maternal lineages. Additionally, we typed the mitochondrial control region (which includes HVR I and II) as an ancestry marker, because it is subjected to low selective pressures because it is non-coding, with its main function being mtDNA replication. These analyses indicate that a number of lineages are represented in this population, with haplogroups B (found mainly in East Asia), E (common among Malaysian people), F (being a widespread Asian haplogroup found in high frequencies in Asia and Japan), and Q (having its highest frequency in Oceania), being the most common. The pattern of mtDNA variation is somewhat different from that of the Y-chromosome. The most common Y-chromosome haplogroup was R, which is most common in Western Asia. Similar findings were seen in the analysis of the whole exome data, in that the most common groupings were Oceanic (Melanesian and Papuan) and Eastern Asian (specifically Han Chinese).

More recently, a similar study was performed on the population of West Timor, the population inhabiting the other side of the island, by Tumonggor et al. (2014). Like their study, we found slightly different haplogroups, and thus origins, of paternal and maternal lineages. Both studies observed the highest frequencies of B mtDNA haplogroups (24.4% in the West Timorese and 21% in the East Timorese), followed by F, Q, and E, while the Y-STR haplogroups differed between studies. Unlike their study, the most common Y-STR haplogroup in the current study was R (30%), while in the West Timorese it was C (26.6%). Using autosomal markers, although through different methodology (whole exome sequencing vs. 37 ancestry informative markers), our analysis showed similar results to what was found in the West Timorese: there are strong Han Chinese and Papuan lineages.

Our study has several limitations. Although our analysis of Y-chromosome and mtDNA markers allowed robust interpretation of the ancestral origins of the East Timorese, genotype information for a much larger number of autosomal markers is needed to do a thorough population admixture analysis. In addition, our sample included too few AMD subjects to make definitive conclusions about the frequencies of established AMD risk variants in AMD subjects in East Timor. Nonetheless, the low prevalence of AMD in this community is explained in part by the very low frequency of several AMD risk variants that are prevalent in other populations throughout the world.

The difference in allele frequencies between the Timorese population and the other genotyped populations, along with the ancestry analysis, show the genetic diversity of this population. Their reduced frequency of deleterious alleles and increased frequency of protective alleles for AMD-associated variants gives reason for their low prevalence of disease. Their ancestry analysis shows they are closely related to Eastern Asians, who also have low prevalence of AMD.

## Author contributions

MM, JR, DS, and MD were involved in the conception and design of the work. JR, JA, GB, LL, SW, FW, ET, JM, IK, KP, DS, and MD contributed to the acquisition of data for analysis. MM, TM, JR, SS, SE, CS, LP, FM, CD, KM, RR, DM, GB, FZ, JW, IK, KP, LF, J B-W, DS, MD were responsible for analysis and/or interpretation of data. MM, TM, SS, SE, CS, LP, FM, JA, CD, KM, RR, DM, SW, EE, KP, and MD were involved in the drafting the manuscript. MM, TM, JR, SS, GB, LL, FZ, JM, IK, JB, LF, DS, and MD were responsible for revising the manuscript critically for important intellectual content. MM, TM, JR, SS, SE, CS, LP, FM, JA, CD, KM, RR, DM, GB, LL, SW, FZ, ET, JW, IK, KP, JB, LF, DS, and MD gave final approval of the version to be published; and agreement to be accountable for all aspects of the work in ensuring that questions related to the accuracy or integrity of any part of the work are appropriately investigated and resolved.

### Conflict of interest statement

The authors declare that the research was conducted in the absence of any commercial or financial relationships that could be construed as a potential conflict of interest.
