# Supplementary material for: Ancestry of the Timorese: age-related macular degeneration associated genotype and allele sharing among human populations from throughout the world
Source: Front Genet. 2015 Jul 9;6:238. doi: 10.3389/fgene.2015.00238 (PMC4496576; doi:10.3389/fgene.2015.00238)

Supplemental Figure 1. Linkage Disequilibrium (r^2) among the genotyped SNPs in AMD-free individuals

1a. Timorese Cohort

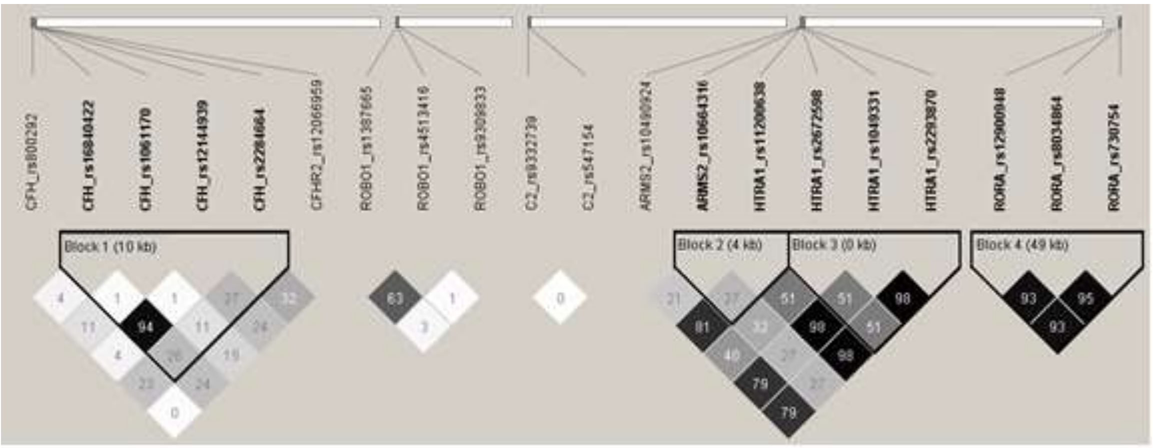

1b. New England Sibpair Cohort

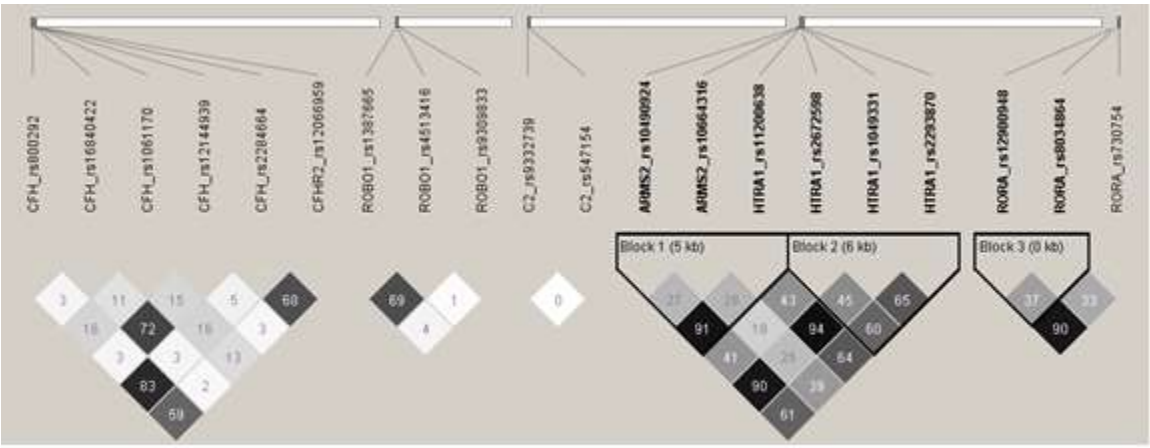

1c. Greek Cohort

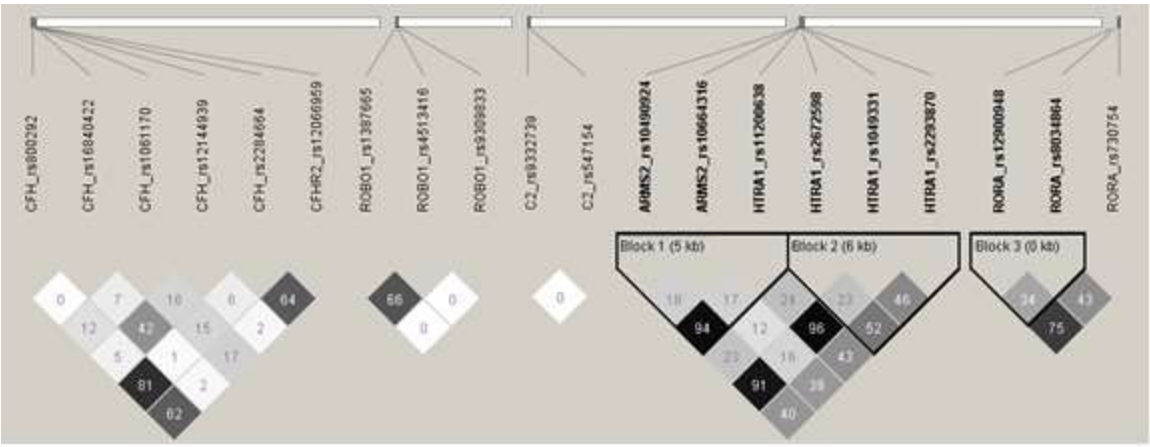

1d. Korean Cohort

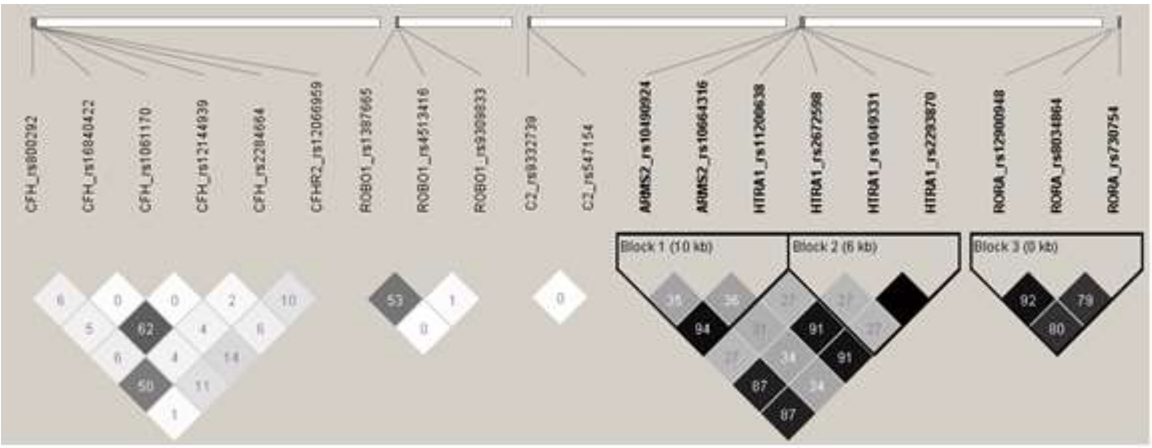

Supplement: Supplementary file 1 [file Image1.PDF]
